# Food insecurity and mental health in South Africa: Structural pathways linking socioeconomic position, behavioural risk factors and comorbidity

**DOI:** 10.1016/j.pmedr.2026.103642

**Published:** 2026-09-21

**Authors:** Lungile Nkosi, Teshager Nigussie, Olalekan A. Ayo-Yusuf

**Affiliations:** Africa Centre for Tobacco Industry Monitoring and Policy Research (ATIM), School of Health Systems and Public Health, University of Pretoria, Gauteng, South Africa

**Keywords:** Food insecurity, Mental health, Comorbidity, Structural equation modelling, South Africa, Socioeconomic status, Nicotine use

## Abstract

**Objective:**

To examine the prevalence of food insecurity in South Africa and the structural pathways linking it with mental health, hypertension-diabetes comorbidity, and socioeconomic and behavioural factors.

**Methods:**

We analysed nationally representative data from 3095 South African adults aged ≥16 years from the South African Social Attitudes Survey conducted in 2025. Survey-weighted generalized structural equation modelling estimated direct, indirect, and reciprocal associations between food insecurity, mental illness, socioeconomic status (SES), nicotine use, alcohol use, and hypertension-diabetes comorbidity.

**Results:**

Overall, 37.4% (95% CI: 34.5, 40.5) of adults reported household food insecurity. Mental illness was strongly associated with food insecurity (OR = 2.82, 95% CI: 1.54, 5.18), while the reciprocal pathway from food insecurity to mental illness was also statistically significant. Higher SES was protective (total effect OR = 0.40, 95% CI: 0.18, 0.86). Nicotine use showed direct associations with food insecurity, whereas alcohol use operated mainly indirectly through nicotine use. Hypertension-diabetes comorbidity was not independently associated with food insecurity.

**Conclusions:**

Although this cross-sectional study cannot infer causal direction, the findings are consistent with reciprocal relationships between food insecurity and mental health within broader socioeconomic and behavioural pathways. Integrated socioeconomic, mental health, and behavioural interventions are needed to reduce food insecurity.

## Highlights

• Food insecurity affected more than one-third of South African adults.
• Mental illness and food insecurity showed strong reciprocal associations.
• Socioeconomic advantage was strongly protective against food insecurity.
• Alcohol was indirectly linked to food insecurity through behavioural pathways.
• Integrated structural, mental health and behavioural responses are needed.

## 1 Introduction

Food insecurity remains a major public health challenge affecting more than 2.3 billion people worldwide, with the greatest burden occurring in low- and middle-income countries (FAO et al., 2024). In South Africa, moderate-to-severe household food insecurity increased from 15.8% in 2019 to 19.7% in 2023, while severe food insecurity rose from 6.4% to 8.0% (Statistics South Africa, 2025a). These patterns occur amid persistent structural pressures, including high unemployment (32.9% nationally, and 46.1% among youth), rising food prices, and climate-related disruptions to food systems (Statistics South Africa, 2025b; Ndlovu and Zenda, 2024; Statistics South Africa, 2025c). Beyond its nutritional consequences, food insecurity has been associated with adverse physical and mental health outcomes, reduced quality of life and increased healthcare utilisation (Gundersen and Ziliak, 2015; Trudell et al., 2021).

Food insecurity and poor mental health frequently coexist, with consistent associations reported for depression, anxiety, psychological distress and reduced well-being (Gundersen and Ziliak, 2015; Trudell et al., 2021). Food insecurity may contribute to poor mental health through chronic stress, uncertainty, social stigma and inadequate dietary quality, whereas poor mental health may reduce educational attainment, employment opportunities, income generation and the capacity to secure adequate food (Jones, 2017; Lund et al., 2010). South African evidence has similarly demonstrated associations between food insecurity, poor mental health and multimorbidity, highlighting these interrelationships within socially disadvantaged populations (Gafari et al., 2026).

Behavioural risk factors may also contribute to these relationships. Tobacco and alcohol use are more common among people experiencing food insecurity and psychological distress, particularly among socioeconomically disadvantaged populations (Gundersen and Ziliak, 2015; Trudell et al., 2021). Nicotine and alcohol use may represent maladaptive coping responses to chronic stress while also competing with household expenditure on food and other basic needs (Lund et al., 2010; Sorsdahl et al., 2012). Food insecurity may in turn reinforce substance use through stress-related coping. Recent South African evidence that polyuse of nicotine products and alcohol was independently associated with food insecurity further supports consideration of behavioural pathways (Nkosi and Ayo-Yusuf, 2026).

Chronic diseases such as hypertension and diabetes may also contribute to food insecurity through increased healthcare expenditure, reduced productivity, and competing household financial demands, while food insecurity may compromise chronic disease management through poorer diet, medication non-adherence and reduced healthcare access (Seligman et al., 2010; Berkowitz et al., 2018; Johnson et al., 2021; Garcia et al., 2018). South African evidence has similarly demonstrated associations between food insecurity, multimorbidity and poor mental health (Gafari et al., 2026; Khofi et al., 2025). These interrelationships support consideration of chronic disease burden alongside socioeconomic, mental health and behavioural factors.

Previous studies have commonly examined these relationships using conventional regression approaches that evaluate individual associations rather than the interconnected pathways through which socioeconomic, behavioural and health factors may jointly influence food insecurity and mental health (Abrahams and Lund, 2022; Shepherd, 2022; Dimba et al., 2025; Dlamini et al., 2024). This study therefore used nationally representative South African data and generalized structural equation modelling (GSEM) to examine direct, indirect, and reciprocal associations between food insecurity, mental health, socioeconomic status, behavioural risk factors, and hypertension-diabetes comorbidity.

## 2 Methods

### 2.1 Study design and population

We analysed data from the South African Social Attitudes Survey (SASAS) conducted between January and March 2025. The SASAS is a nationally representative, cross-sectional, face-to-face household survey of individuals ≥16 years (Human Sciences Research Council, 2020).

SASAS employs a multi-stage stratified probability sampling design. The multi-stage probability sample was stratified by province, geographic location, and population group. Enumeration areas were selected with probability proportional to size, households were randomly selected within enumeration areas, and one eligible respondent was selected within each household using a Kish grid. Of 3095 participants, 2979 (96.3%) had complete data for all model variables and were included in the regression and GSEM analyses.

Survey weights were applied to account for clustering, stratification, unequal probabilities of selection, and non-response.

### 2.2 Measures

Age was categorized as 16–34, 35–54, and ≥ 55 years. Sex was based on self-report (“male” or “female”). Self-identified race was categorized as Black African, Coloured, Indian/Asian, or White. Marital status was classified as currently married, never married, or previously married. Educational attainment was grouped as less than high school, completed high school, or more than high school. Employment status was categorized as employed, unemployed, or not economically active. Residence was classified as urban or rural.

Socioeconomic status (SES) was modelled as a latent construct indicated by educational attainment, employment status, and household asset ownership. Household assets were summarized using principal component analysis, and the first component was categorized into tertiles. Higher values reflected greater socioeconomic position.

Food insecurity was measured using the item: *“In the past 12 months, was there any time you have run out of food and not been able to purchase more?”* Respondents answering “yes” were classified as food insecure (McKechnie et al., 2018). This single-item measure has been used in South African population surveys as a pragmatic indicator of household food-related hardship, although it does not capture the multidimensionality or severity levels of food insecurity (Nkosi and Ayo-Yusuf, 2026).

Mental illness was defined as a self-reported previous diagnosis by a health professional of mental illness, including stress, depression, or emotional problems. Emotional well-being was assessed using the three-item hedonic (emotional well-being) domain of the Mental Health Continuum–Short Form (MHC-SF), covering happiness, interest in life and life satisfaction (Keyes et al., 2008; Keyes, 2002). The MHC-SF has demonstrated good validity and reliability in South African populations (Keyes et al., 2008). Higher scores indicated better emotional well-being, and internal consistency was high in this study (Cronbach's α = 0.89).

Current nicotine use was defined as self-reported use (“every day” or “some days”) of any tobacco or nicotine product.

Problematic alcohol use was measured using the four-item CAGE instrument. A score ≥ 2 was classified as indicative of problem drinking. Alcohol use was categorized as never drinker, non-problematic drinker, or problematic drinker.

Both variables were included because previous evidence suggests they may function as behavioural responses to chronic socioeconomic stress while simultaneously contributing to household financial strain and food insecurity (Lund et al., 2010; Nkosi and Ayo-Yusuf, 2026).

Participants were asked whether they had ever been diagnosed by a health professional with hypertension or diabetes. Comorbidity was defined as the co-occurrence of both conditions. Hypertension-diabetes comorbidity was included to examine whether chronic disease burden contributed independently to food insecurity after accounting for socioeconomic and behavioural pathways.

### 2.3 Statistical analysis

Weighted prevalence estimates of food insecurity were calculated overall and across sociodemographic and health-related variables. Design-based chi-square tests were used to assess bivariate associations. Survey weights and design variables were applied to account for clustering, stratification, differential selection probabilities, and non-response, thereby ensuring that the results are generalizable to the South African adult population.

As a sensitivity analysis, diagnosed mental illness in the fully adjusted Poisson regression was replaced with the continuous emotional well-being score to assess whether the association with food insecurity was robust to an alternative measure of mental health.

Generalized structural equation modelling (GSEM) with non-recursive paths was used to examine theoretically hypothesised reciprocal associations among food insecurity, mental illness and hypertension-diabetes comorbidity while accounting for socioeconomic and behavioural pathways. Food insecurity and mental illness were modelled as binary endogenous variables and comorbidity as an ordinal endogenous variable (none, one or both conditions). Nicotine use and emotional well-being were also endogenous variables, while alcohol use and sociodemographic characteristics were exogenous predictors. SES was modelled as a latent construct indicated by educational attainment, employment status, and household asset ownership. Binary outcomes were modelled using logit links, and continuous outcomes using identity links with Gaussian distributions.

Model identification was supported by exclusion restrictions whereby selected exogenous predictors entered specific endogenous equations but were excluded from corresponding equations in the non-recursive system, together with identification constraints for the latent SES construct. Exogenous predictors were assumed to be uncorrelated with structural errors. Parameters were estimated simultaneously using survey-weighted maximum likelihood with robust standard errors. Local stability of the feedback system was supported by a spectral radius of 0.217 (<1) (Bentler and Freeman, 1983). Reciprocal paths represent simultaneous associations and not temporal or causal effects. Complete-case analysis included 2979 participants (96.3%); 116 (3.7%) with missing data on one or more model variables were excluded.

Direct, indirect, and total effects were estimated within the GSEM framework and presented as odds ratios (ORs) with 95% confidence intervals. Statistical significance was set at *p* < 0.05. Analyses were performed using Stata version 18 (StataCorp LLC, College Station, TX, USA).

The South African Social Attitudes Survey protocol and primary data collection were approved by the Human Sciences Research Council Research Ethics Committee (REC 5/17/08/11), with informed consent obtained from all participants. The protocol for the present secondary analysis of anonymised survey data was approved by the University of Pretoria Faculty of Health Sciences Research Ethics Committee (FHS REC 451/2023). The study was conducted in accordance with the Declaration of Helsinki and applicable South African National Research Ethics Guidelines.

## 3 Results

### 3.1 Participant characteristics and correlates of food insecurity

A total of 3095 respondents were included. Most participants self-identified as Black African (79.3%), 51.9% were female, 68.0% lived in urban areas, and 42.1% were unemployed. A prior mental illness diagnosis was reported by 6.8%, while 37.6% currently used nicotine products. Additional characteristics of the study sample are shown in Table 1.

**Table 1:** Distribution of sociodemographic and behavioural characteristics of South African adults participating in the South African Social Attitudes Survey, 2025 (*N* = 3095).

Variables		Unweighted n	Weighted % (95% CI)
**Race**	Black African	1849	79.3 (75.7, 82.4)
	Coloured	526	9.0 (7.3, 11.1)
	Indian/Asian	356	2.9 (2.2, 3.8)
	White	342	8.8 (6.6, 11.5)
**Age group (years)**	16–34	1233	44.4 (41.1, 47.8)
	35–54	1152	36.8 (33.7, 39.9)
	≥55	696	18.8 (16.7, 21.3)
**Sex**	Male	1460	48.1 (44.9, 51.3)
	Female	1628	51.9 (48.7, 55.1)
**Marital status**	Currently married	843	23.8 (21.1, 26.6)
	Previously married^a^	500	12.1 (10.4, 14.1)
	Never married^b^	1704	64.1 (60.7, 67.4)
**Education**	Less than high school^c^	1039	35.9 (32.8, 39.2)
	High school	1299	44.8 (41.5, 48.2)
	Above high school	742	19.3 (16.7, 22.2)
**Residence**	Rural	799	32.0 (27.7, 36.5)
	Urban	2290	68.0 (63.5, 72.3)
**Employment status**	Unemployed	1075	42.1 (38.7, 45.5)
	Not economically active	946	29.3 (26.5, 32.2)
	Employed	1068	28.6 (25.9, 31.5)

**Self-reported health conditions**			
Mental illness	Not previously diagnosed	2893	93.2 (91.4, 94.7)
	Previously diagnosed	202	6.8 (5.3, 8.6)
Comorbidities	None	2433	79.5 (76.7, 82.0)
	One condition	501	16.0 (13.9, 18.4)
	Two conditions	161	4.5 (3.4,6.0)

**Behavioural risk factors**			
Nicotine use	Non user	1923	62.4 (58.9, 65.8)
	Current nicotine user	1172	37.6 (34.2, 41.1)
Problem drinking	Non-alcohol users	2063	65.2 (62.5, 67.9)
	No problem drinking	779	24.7 (22.4, 27.2)
	Problem drinking	253	10.0 (8.4, 12.0)

Note: All percentages (%) are weighted, and all raw counts (n) are unweighted

^a^Previously married includes divorced, widowed, or separated

^b^Never married includes both those who are engaged and those not engaged

^c^Includes those with no school, less than primary school, and those with some high school education

^d^Includes those retired, homemakers, or very disabled and unable to work, or attending school

Overall, 37.4% (95% CI: 34.5, 40.5) of participants experienced food insecurity. Prevalence was significantly higher among Black African and Coloured participants, those with lower SES, unemployed participants, those reporting mental illness and current nicotine product users. Food insecurity was also more prevalent among participants with hypertension-diabetes comorbidity (Table 2).

**Table 2:** Distribution of food insecurity by sociodemographic characteristics and behavioural characteristics among South African adults aged 16 years and older participating in the South African Social Attitudes Survey, 2025.

Characteristics	Food insecurity^a^		
	n^b^	Weighted %^c^	*p*-value*
**Total**	995	37.4	–
**Race**			<0.01
Black African	759	42.8	
Coloured	152	23.8	
Indian/Asian	48	11.0	
White	29	8.3	
**Age (years)**			0.32
16–34	426	36.3	
35–54	380	40.4	
≥55	185	34.4	
**Gender**			<0.01
Male	409	32.3	
Female	584	42.1	
**Education**			<0.01
< High school^d^	470	46.9	
Completed high school	416	39.7	
> High school	105	15.0	
**Marital status**			<0.01
Never married^e^	627	40.8	
Previously married^f^	140	30.3	
Currently married	213	31.2	
**Employment status**			<0.01
Unemployed	487	50.3	
Not economically active^g^	283	32.8	
Employed	223	23.2	
**Area type**			0.06
Rural	376	51.6	
Urban	617	30.8	
**Mental illness**			0.01
No	918	36.3	
Yes	77	53.0	
**Comorbidities**^**h**^			0.01
None	756	36.5	
One condition	191	45.4	
Two conditions	46	25.6	
**Alcohol use**			<0.01
Non-alcohol users	340	34.9	
No problem drinking	542	37.1	
Problem drinking	113	47.9	
**Nicotine Use**			0.02
Non-user	568	33.8	
Current nicotine user	427	41.9	

**Notes**:*P-values were obtained from design-based chi-square tests accounting for the complex survey design.

^a^Food insecurity was defined as those who ran out of food and not been able to purchase more in the past year

^b^n = unweighted count of those who are food insecure

^c^Weighted %  = proportion food insecure, weighted adjusted for complex survey design.

^d^Includes those with no school, less than primary school, and those with some high school education

^e^Never married includes both those who are engaged and those not engaged

^f^Previously married includes divorced, widowed, or separated

^g^Includes those retired, homemakers, or very disabled and unable to work, or attending school

^h^Includes Hypertension and Diabetes

The sensitivity analysis using continuous emotional well-being provided consistent evidence of the association with mental health: food-insecure participants had lower mean emotional well-being than food-secure participants (8.0 vs 9.1; Wald F = 34.4, *p* < 0.01), with the direction of association remaining consistent in the fully adjusted Poisson model.

### 3.2 Structural model findings

The GSEM identified interrelated associations among socioeconomic, behavioural and health factors and food insecurity (Fig. 1). The fully fitted model with detailed path coefficients and odds ratios is presented in Supplementary Fig. S1.

**Fig. 1:**
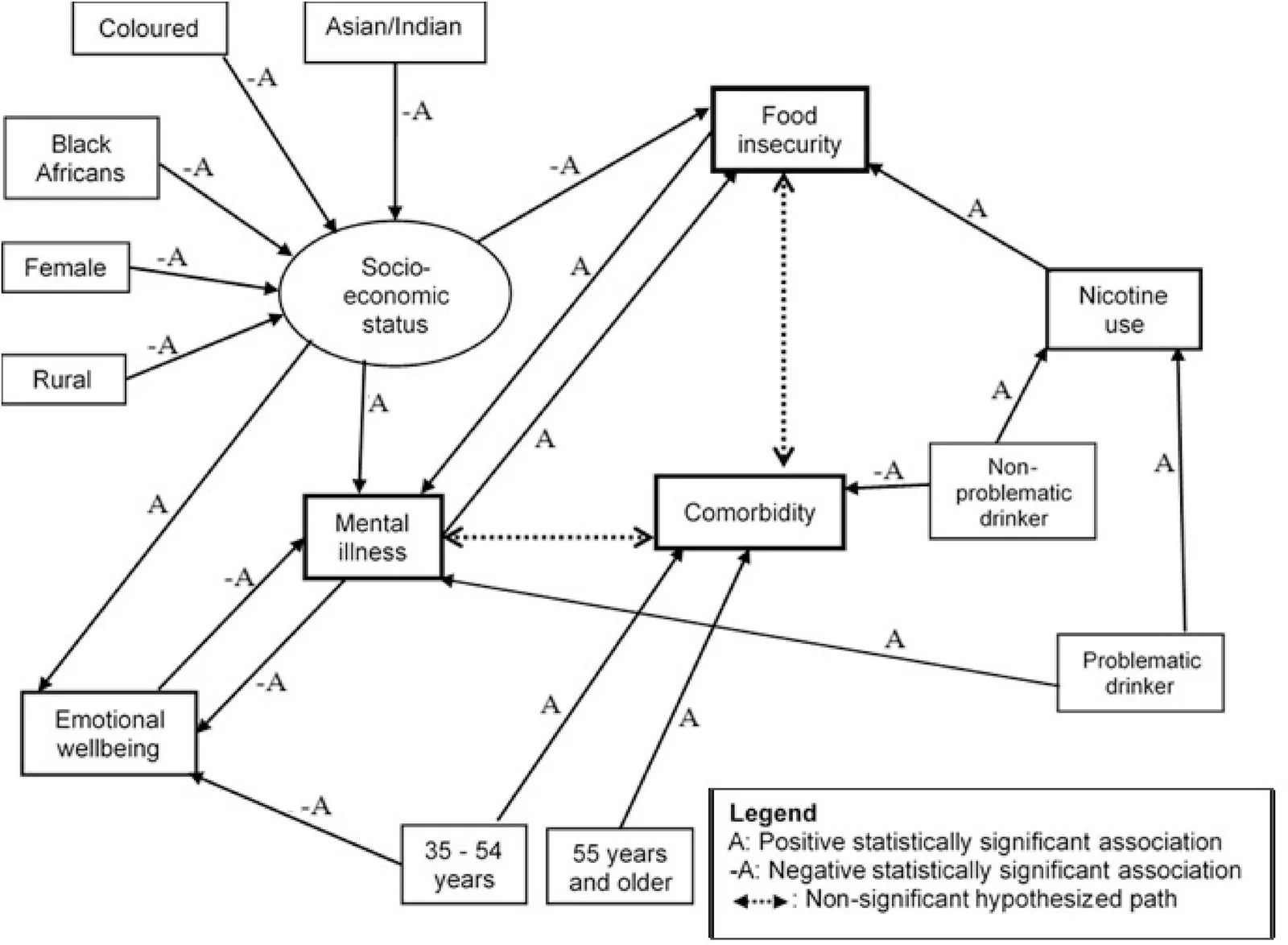
Simplified generalized structural equation model of associations between food insecurity, mental illness and comorbidity among South African adults, 2025.

The GSEM showed reciprocal positive associations between food insecurity and mental illness (Fig. 1). Mental illness was associated with greater odds of food insecurity (OR = 2.82; 95% CI: 1.54, 5.18), while nicotine use was also directly associated with food insecurity (OR = 1.64; 95% CI: 1.18, 2.28) (Table 3; Fig. S1). Higher SES was inversely associated with food insecurity. Reciprocal associations involving hypertension-diabetes comorbidity were not statistically significant (Table 3; Fig. 1).

**Table 3:** Direct and total association with food insecurity from generalized structural equation modelling among South African adults, 2025**.**

Predictor	Direct OR (95% CI)	Total OR (95% CI)
Mental illness (Yes vs No)	2.82 (1.54, 5.18)	2.80 (1.51, 5.17)
Hypertension–Diabetes Comorbidity	0.97 (0.73, 1.30)	1.14 (0.72, 1.80)
Nicotine use (Current vs non-user)	1.64 (1.18, 2.28)	2.00 (1.01, 3.95)
Socioeconomic Status (Higher/Better off)	0.17 (0.10, 0.30)	0.40 (0.18, 0.86)
Alcohol use (Ref: Never drinker)		
Non-problematic drinker	0.97 (0.69, 1.35)	4.50 (1.66, 12.22)
Problematic drinker	1.38 (0.83, 2.29)	12.56 (2.72, 57.87)

Notes

• OR = Odds Ratio; CI = Confidence Interval.

• Direct effects represent associations with food insecurity adjusting for mediators.

• Total effects represent the sum of direct and indirect effects estimated in the GSEM.

• Full mediator-specific indirect pathways are presented in Supplementary Table S1.

Alcohol had no significant direct association with food insecurity, but showed significant indirect associations through nicotine use and through mental illness (Table S1; Fig. 1). Higher SES retained a strong protective total association with food insecurity (OR = 0.40, 95% CI: 0.18, 0.86) (Table 3), reflecting both opposing direct and indirect pathways.

The total associations of mental illness and current nicotine use with food insecurity remained statistically significant. Alcohol use also showed a significant total association with food insecurity despite a non-significant direct association (Table 3).

The pathways involving SES showed that higher SES was directly associated with lower odds of food insecurity but was also associated with higher odds of mental illness, which in turn was positively associated with food insecurity. Thus, the overall protective association of SES with food insecurity reflected a strong direct pathway partly offset by an indirect pathway through mental illness (Fig. 1; Table S1).

## 4 Discussion

This study provides nationally representative evidence that food insecurity in South Africa is interrelated with mental illness, socioeconomic disadvantage and behavioural risk factors through multiple direct and indirect pathways. The GSEM showed reciprocal positive associations between food insecurity and mental illness, a strong protective total association of higher SES, and direct and indirect behavioural pathways involving nicotine and alcohol use. In contrast, hypertension-diabetes comorbidity was not independently associated with food insecurity after accounting for these interconnected factors.

The reciprocal positive associations between food insecurity and mental illness are consistent with evidence that food insecurity may adversely affect mental health through chronic stress, uncertainty and material deprivation, while poor mental health may impair employment, income generation and the capacity to secure adequate food (Gundersen and Ziliak, 2015; Trudell et al., 2021; Jones, 2017; Gafari et al., 2026; Khofi et al., 2026). However, these simultaneously estimated associations should not be interpreted as evidence of temporal or causal bidirectionality given the cross-sectional design.

The sensitivity analysis further supported the relationship between food insecurity and mental health: food-insecure participants had significantly lower emotional well-being, and the association remained in the fully adjusted Poisson model. This suggests that the finding was not dependent solely on the binary indicator of diagnosed mental illness.

The positive association between higher SES and diagnosed mental illness contrasts with the established social gradient in mental health burden and may partly reflect greater access to recognition and diagnosis among socioeconomically advantaged groups, particularly as higher SES was concurrently associated with better emotional well-being (Gordon et al., 2020; Myer et al., 2008).

In contrast to the mental health findings, although hypertension-diabetes comorbidity was associated with food insecurity in unadjusted analyses, this association was attenuated after accounting for socioeconomic position, behavioural risk factors and mental illness. This suggests that the relationship between chronic disease burden and food insecurity may largely reflect shared structural determinants rather than an independent effect of chronic disease itself. Recent South African evidence similarly shows that multimorbidity and food insecurity frequently coexist within contexts of socioeconomic disadvantage (Gafari et al., 2026).

The financial consequences of chronic disease may nevertheless operate over longer periods and may not be fully captured cross-sectionally. In South Africa, even where chronic disease care is provided through the public healthcare system, patients may incur direct and indirect costs, including transport costs and productivity losses (Mutyambizi et al., 2019; Kohli-Lynch et al., 2022).

Behavioural pathways were also evident: nicotine use was directly associated with food insecurity, while alcohol operated indirectly through nicotine use and mental illness. These pathways may reflect clustering of behavioural risks in response to socioeconomic and psychological stress and competition between expenditure on addictive products and essential household needs. Recent South African cross-sectional and longitudinal evidence similarly links food insecurity with nicotine/tobacco and alcohol use (Nkosi and Ayo-Yusuf, 2026; Pengpid and Peltzer, 2025). Behavioural risk factors may therefore both reflect and reinforce vulnerability to food insecurity.

The observed racial differences should not be interpreted as reflecting intrinsic characteristics associated with race. Rather, race in South Africa functions as a marker of historically produced structural inequalities arising from apartheid-era policies and their enduring effects on residential location, education, employment, wealth accumulation and access to economic resources (Branson et al., 2024; Pellicer and Ranchhod, 2023). The observed associations therefore likely reflect enduring structural inequities rather than race itself.

The findings support addressing food insecurity within its broader socioeconomic context. Policies strengthening household economic and food security, alongside integration of mental health and behavioural risk assessment into social support programmes, may help address mutually reinforcing vulnerabilities.

Strengths of this study include the nationally representative sample, use of GSEM to examine interconnected pathways, and sensitivity analysis using emotional well-being.

Several limitations should be considered. First, the cross-sectional design precludes establishing temporal or causal direction, and reciprocal GSEM associations should therefore not be interpreted as causal bidirectionality. Second, food insecurity was assessed using a single-item indicator that does not capture its multidimensionality or distinguish moderate from severe food insecurity. Mental illness was based on self-reported prior diagnosis and may therefore reflect both underlying morbidity and access to diagnosis. Complete-case analysis may have introduced selection bias because missingness was associated with sociodemographic characteristics, although only 3.7% of observations were excluded. Finally, self-reported behavioural and health measures may be affected by recall or reporting bias.

## 5 Conclusion

Food insecurity in South Africa is closely interrelated with mental health within broader socioeconomic and behavioural pathways. Although reciprocal associations between food insecurity and mental illness were observed, the cross-sectional design precludes conclusions about causal direction. The attenuation of associations with chronic disease burden after accounting for socioeconomic, behavioural and mental health factors suggests an important role for shared structural vulnerabilities. Integrated interventions addressing socioeconomic disadvantage, mental health and behavioural risk factors may help reduce food insecurity and its associated health consequences.

## Generative AI and AI-assisted technologies

During the preparation of this manuscript, the authors used ChatGPT (OpenAI, San Francisco, CA, USA) and Grok (xAI Corp., San Francisco, CA, USA) for editorial purposes. All analytical content and interpretation are original. The authors reviewed and edited all outputs and take full responsibility for the content of the published article.

## CRediT authorship contribution statement

**Lungile Nkosi:** Writing – review & editing, Writing – original draft, Validation, Project administration, Methodology, Formal analysis, Conceptualization, Data curation. **Teshager Nigussie:** Writing – review & editing, Visualization, Validation, Methodology, Formal analysis, Data curation. **Olalekan A. Ayo-Yusuf:** Writing – review & editing, Supervision, Resources, Methodology, Funding acquisition, Data curation, Conceptualization.

## Informed consent statement

The South African Social Attitudes Survey (SASAS), conducted in 2025, was undertaken under a protocol approved by the Human Sciences Research Council Research Ethics Committee (REC 5/17/08/11), and all participants provided informed consent before completing the survey. The protocol for the present secondary analysis of anonymised SASAS data was approved by the University of Pretoria Faculty of Health Sciences Research Ethics Committee (FHS REC 451/2023). The study was conducted in accordance with the Declaration of Helsinki and applicable South African National Research Ethics Guidelines.

## Funding

This study was supported by the Africa Capacity Building Foundation (ACBF) through Grant No. 337, with funding support from the Gates Foundation. The funding covered data acquisition only; no funding was provided for data analysis, manuscript preparation, or publication. The conclusions and opinions expressed in this work are those of the authors alone and shall not be attributed to the Foundation.

## Declaration of competing interest

The authors declare the following financial interests/personal relationships which may be considered as potential competing interests: Olalekan Ayo-Yusuf reports financial support was provided by African Capacity Building Foundation. If there are other authors, they declare that they have no known competing financial interests or personal relationships that could have appeared to influence the work reported in this paper.

## Data Availability

The data supporting the findings of this study are available from the principal investigator, Prof. Olalekan Ayo-Yusuf, upon reasonable request.

## Appendix A Supplementary data

Supplementary material Table S1. Indirect effect estimates of socioeconomic, behavioural, mental illness, and comorbidity pathways to food insecurity among South African adults, 2025, from generalized structural equation modelling. Figure S1 remains unchanged.
