# Supplementary material for: Food insecurity and mental health in South Africa: Structural pathways linking socioeconomic position, behavioural risk factors and comorbidity
Source: Prev Med Rep. 2026 Sep 21;71:103642. doi: 10.1016/j.pmedr.2026.103642 (PMC13634340; doi:10.1016/j.pmedr.2026.103642)
Supplement: Supplementary file 1 [file mmc1.docx]

**Table S1.** **Indirect effect estimates of socioeconomic, behavioural, mental illness and comorbidity pathways to food insecurity among South African adults, 2025, from generalized structural equation modelling.**

| **Predictor** | **Mediator** | **OR (95% CI)** |
| --- | --- | --- |
| **Mental illness → Food insecurity** | Comorbidity | 0.99 (0.90, 1.09) |
| Reciprocal path | Comorbidity | 1.01 (0.95, 1.07) |
|  |  |  |
| **Comorbidity → Food insecurity** | Mental illness | 1.17 (0.79, 1.76) |
| Reciprocal path | Mental illness | 1.39 (0.82, 2.35) |
|  |  |  |
| **Nicotine use → Food insecurity** | Mental illness | 1.21 (0.68, 2.16) |
|  | Comorbidity | 1.01 (0.94, 1.07) |
|  | **Total indirect** | 1.22 (0.68, 2.19) |
|  |  |  |
| **Socioeconomic Status → Food insecurity** | Mental illness | 2.22 (0.92, 5.36) |
|  | Comorbidity | 1.02 (0.93, 1.08) |
|  | Nicotine use | 1.05 (0.93, 1.19) |
|  | **Total indirect** | 2.35 (0.96, 5.79) |
|  |  |  |
| **Alcohol use (Non-problematic) → Food insecurity** | Nicotine use | 2.64 (1.36, 5.11) |
|  | Mental illness | 1.74 (0.79, 3.85) |
|  | Comorbidity | 1.01 (0.89, 1.15) |
|  | **Total indirect** | 4.65 (1.72, 12.53) |
|  |  |  |
| **Alcohol use (Problematic) → Food insecurity** | Nicotine use | 2.88 (1.37, 6.02) |
|  | Mental illness | 3.18 (0.92, 10.99) |
|  | Comorbidity | 1.00 (0.95, 1.04) |
|  | **Total indirect** | 9.12 (2.08, 40.08) |
|  |  |  |
| **Female → Food insecurity** | SES | 1.32 (1.09, 1.61) |
| **Rural → Food insecurity** | SES | 2.08 (1.63, 2.66) |
| **Black African → Food insecurity** | SES | 11.52 (5.66, 23.36) |
| **Coloured → Food insecurity** | SES | 8.18 (4.31, 15.57) |
| **Indian/Asian → Food insecurity** | SES | 2.28 (1.45, 3.60) |
| **Never married → Food insecurity** | SES | 1.19 (0.98, 1.44) |
| **Previously married → Food insecurity** | SES | 1.06 (0.85, 1.33) |

SES = Socioeconomic status

### OR = Odds Ratio; CI = Confidence Interval.

**Figure S1: Generalized structural equation modelling path diagram of socioeconomic, behavioural and health associations with food insecurity among South African adults, 2025.**

*NB: Categorical (binary/ordinal) endogenous outcomes (food insecurity, mental illness status, comorbidity) are shown in red rectangles; mediator variables (mental well-being score, current nicotine use status) are shown in green rectangles; the latent variable Socioeconomic status is depicted as a purple oval. Socioeconomic status was a latent variable measured using three ordinal indicators (education level, employment status, household assets) with a probit link. Arrows represent hypothesized directional effects:* ***Blue*** *= positive and statistically significant (p < 0.05);* ***Orange****: negative and statistically significant (p < 0.05);* ***Yellow:*** *factor loadings of factors on the latent SES construct to its indicators (education level = 1.0, employment status= 0.72, household assets = 1.85);* ***Black:*** *non-significant hypothesized retained for theoretical completeness. Bidirectional arrows indicate non-recursive associations. Path coefficients with the 95% confidence intervals of the associations in the non-recursive system are interpreted as linear associations (continuous endogenous variables such as emotional well-being), log-odds (for binary/ordinal outcomes with logit links, including mental illness, food insecurity, and comorbidity), and loadings (measurement model-SES). Estimates are from weighted robust maximum likelihood with non-recursive paths among endogenous variables.*
